# A Dynamic Part-Attention Model for Person Re-Identification

**DOI:** 10.3390/s19092080

**Published:** 2019-05-05

**Authors:** Ziying Yao, Xinkai Wu, Zhongxia Xiong, Yalong Ma

**Affiliations:** 1 School of Transportation Science and Engineering, Beihang University, Beijing 100191, China; zyyao@buaa.edu.cn (Z.Y.); xinkaiwu@buaa.edu.cn (X.W.); mayalong@buaa.edu.cn (Y.M.); 2 Beijing Advanced Innovation Center for Big Data and Brain Computing, Beihang University, Beijing 100191, China

**Keywords:** person re-identification, cross cameras, convolutional neural network, attention parts, dynamic combination

## Abstract

Person re-identification (ReID) is gaining more attention due to its important applications in pedestrian tracking and security prevention. Recently developed part-based methods have proven beneficial for stronger and explicit feature descriptions, but how to find real significant parts and reduce miscorrelation between images to improve accuracy of ReID still leaves much room to improve. In this paper, we propose a dynamic part-attention (DPA) method based on masks, which aims to improve the use of variable attention parts. Particularly, a two-branch network with a dynamic loss function is designed to extract features of the global image and the parts of the body separately. With the comprehensive but targeting learning strategy, the proposed method can capture discriminative features based, but not depending on, masks, which guides the whole network to focus on body features more consciously and achieves more robust performance. Our method achieves rank-1 accuracy of 91.68% on public dataset Market1501, and experimental results on three public datasets indicate that the proposed method is effective and achieves favorable accuracy when compared with the state-of-the-art methods.

## 1. Introduction

With the wide applications of surveillance cameras, person re-identification (ReID) has become increasingly important recently. ReID is a widely used term and a specific task in computer vision attracting rapidly increased attentions. ReID means to recognize the same person under different conditions, and it is widely considered to be a sub-question for image retrieval, targeting matching images of the probe person across multiple cameras and scenes. Therefore, it is critical for surveillance applications such as pedestrian tracking and security prevention. For instance, when given a monitored pedestrian image, it aims to tell whether the person was observed in another place or time. Due to its great value, person ReID has become a hot research focus for both academia and industries. Although many efforts have been dedicated to this problem, person ReID is still facing two major challenges. The first challenge is caused by various appearance of person, including poses, resolution, illumination, and camera views; and second one comes from cluttered environment, including occlusion, fusion, etc. To address both two challenges, many methods [1,2] directly learn features from the whole image containing both the body and background. However, through these methods, unnecessary noises have been taken into the learning process, resulting in unsatisfying precision. In recent years, some researchers have suggested tackling these issues through parts learning. For example, methods [3,4,5,6] split the whole image into several same size parts in order to focus on local features and then connect each part at the end. These part-based methods have proven more effective than those only using global information, indicating that focusing on certain parts will strengthen the ability of feature description. In addition, there are many cases that persons are captured partially by CCTV cameras because of occlusion, so it is of great significance in surveillance applications to improve the accuracy of part-based ReID methods.

**Figure 1:**
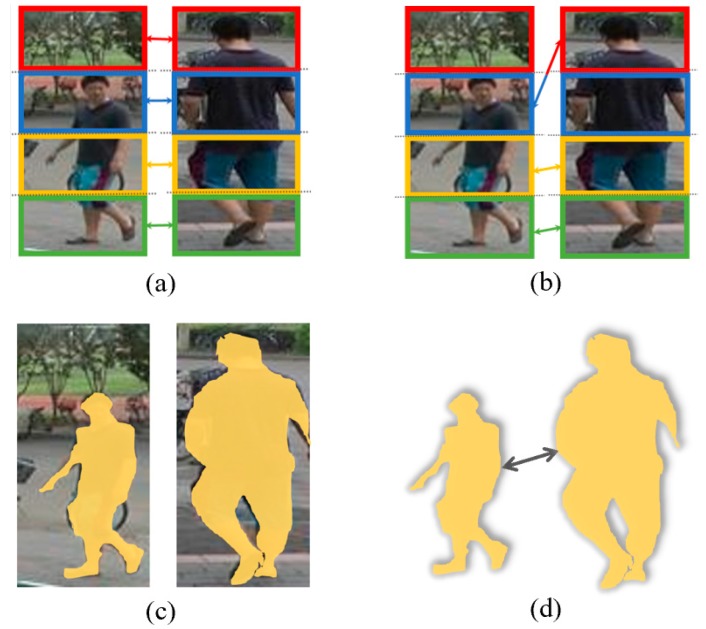
Comparison of part-based methods: (**a**) Dividing images into rectangle parts; (**b**) part alignment model; (**c**) proposed dynamic and accurate attention parts based on body regions; and (**d**) explicitly and stable correspondence between parts.

Although part-learning methods have proven beneficial to the performance of ReID, how to find real discriminative parts among the whole image is still a challenging issue. As mentioned above, many methods divide the image into several equal-size parts commonly four or six and then combine them at the end, as shown in Figure 1a. However, this rigid dividing strategy clearly damages the continuity of the whole image and the misalignment among parts could further bring obstacles for feature matching, therefore downgrading the effectiveness of each part. Some works commit to alignment algorithm between corresponding parts, as shown in Figure 1b, using external pose estimation [7,8] or less supervision transition [5,9]. However, this type of methods is error-prone and easy to be out of control. Furthermore, most of existing methods select parts using rectangle boxes, which contains redundant background and less targeted. This motivates us to rethink how to obtain stable and relatively accurate part in part-based learning.

As person ReID is targeting at persons, it is reasonable to put attention on the body regions that contain much information like shape, clothes, accessories, etc. This is similar to the concept of attention mechanism. Following this inspiration, we propose a dynamic part-attention (DPA) model which chooses the body region as part that we put more attention for person ReID, called attention parts in this paper. Here, part-attention comes from attention mechanisms in neural networks. Intuitively, it applies the neural network to focus on a subset of its inputs or features. Part-attention means the body part which deserves more attention than background, and we tell the network which part to focus explicitly by introducing the body mask in the proposed method. As shown in Figure 1c, these body attention parts are explicit and accurate, so they can be easily compared with each other and find the corresponding parts in Figure 1d. Furthermore, the selected body regions can be flexibly changed in variable scenes and camera views.

**Figure 2:**
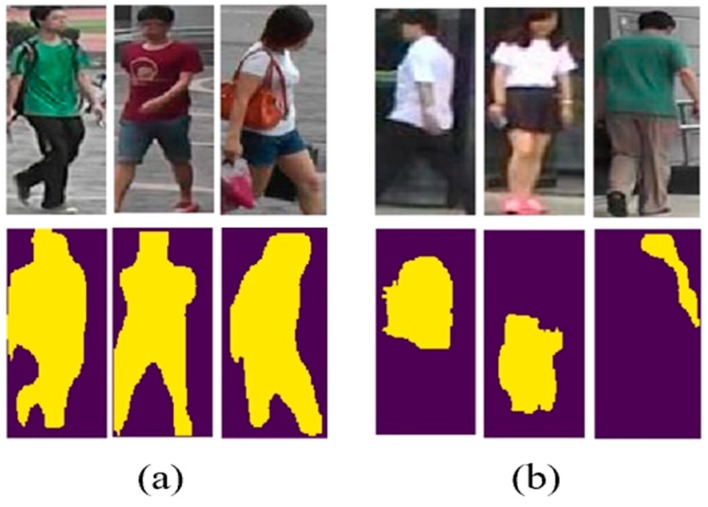
Some results of segmentation masks: (**a**) Complete; and (**b**) incomplete. The top row denotes original images, with corresponding binary masks below, where body regions display in yellow pixels.

To obtain these dynamic attention parts, an intuitive idea is applying segmentation method to segment and obtain masks. With the help of recently developed segmentation methods like Mask R-CNN [10] and FCN [11], we can obtain much more accurate masks conveniently, so we design our DPA model based on body masks. Due to the bias of training datasets between two fields, we might fail to get accurate body masks in many times. As shown in Figure 2, two different body masks were extracted: (a) Complete masks, which cover the majority of the body region; and (b) incomplete masks, which only cover some parts of bodies. Note with the external help of segmentation, even incomplete body masks may contain more significant and discriminative information and deserve attention. Therefore, this motivates us to take a full utilization of all these masks dynamically, including both complete and incomplete ones. Thus, dynamic means that the mask of person body is flexible instead of dividing images into fixed parts, and we utilize them with dynamic combinations to get more accurate and discriminative regions and enable the method based but not depending on masks.

**Figure 3:**
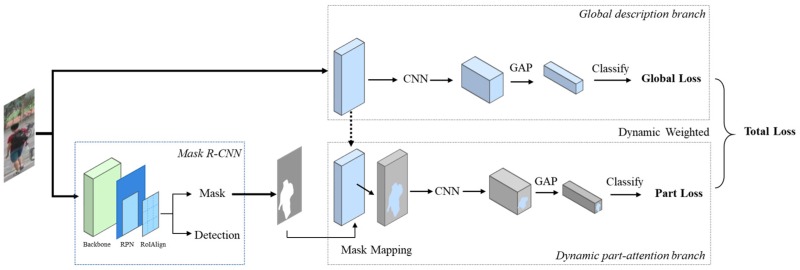
The framework of the proposed dynamic part attention (DPA) method for person ReID. RGB images are the inputs. We first use Mask R-CNN segment body masks. Then the main framework consists of two branches. In the figure, we first choose the ResNet-50 as the backbone to extract features, then design two branches to estimate total loss. The first branch uses global average pooling (GAP) to calculate the global loss, and the second one uses segmented mask as the attention part to calculate the mask-based part loss. Losses from two branches are combined dynamically for training to achieve mutual promotion.

As illustrated in Figure 3, the proposed DPA model essentially consists of two branches which are separated but complementary with each other. In the global branch, we use the raw image as input to get consistent and structured information for a classification work, and help guide the overall learning process. In the part-attention branch, we introduce body masks extracted by segmentation network and saved in early process in order to pay more attention to the body region. It aims to extract more discriminative information for another classification work. At last, we design a dynamic loss combination to integrate information from global and the attention part to achieve mutual and complementary learning state and to help improve robustness of the proposed method. Experiments on three public datasets including Market-1501, DukeMTMC-reID, and CUHK03 confirm effectiveness of the proposed method in comparison with the state-of-the-art methods.

## 2. Related Work

### 2.1. Features Representation

Developing robust and representative features for person images is a key step in person ReID. The selection and representation of image features can be roughly categorized into two types.

First, some researchers focused on designing handcrafted feature descriptors to extract useful information from pedestrian images. Some methods [12,13,14] were proposed to create a color-based descriptor by color space or color names, combined with salient edge or texture histograms to obtain fusion features. However, these handcrafted features require complex calculating procedures and are easily affected by human errors, leading to less satisfying results. 

Second, in recent years, deep learning-based person ReID methods have achieved impressive performance in many large datasets. Many studies choose convolutional neural network (CNN) to extract image features. It consists of several layers to conduct convolutional computation and outputs feature vectors to represent the image abstractly. With the advantage of automatic learning and strong expression, deep frameworks were designed to extract multiple types of features. As described above, deep features for person ReID mainly divide into two types: 1) Global features: Methods in [2,15,16,17,18] use the whole image as input and learn features for both background and foreground, then comparing the similarity among these features; and 2) Part features: Part learning occupies an increasing proportion of recent studies, which focus on more detailed features and spatial information. For example, methods in References [3,6,19] divided images into several equal parts and joined individual parts by concatenating and fuse with global features at the end. Zhang et al. [5] proposed a novel framework to align corresponding body regions between a pair of images, and then fused global and local features into a mutual learning process. Instead of dividing images into same-size parts, some methods localize specific regions to choose flexible parts. For example, Yao et al. chose saliency feature maps as bounding boxes, and then clustered boxes into four parts, and each part followed by ROI pooling is an individual branch for learning. Li et al. [3] introduced Spatial Transformer Networks (STN) into the framework, using two constraints to localize latent parts. Zheng et al. [8] combined extra Pose-Box generated by pose estimation network with the original image and created a fusion pose-based descriptor. These methods have proven effective when addressing more attention on the body region, but the accuracy is affected by messy background. To further improve the accuracy of person ReID, the body mask information was applied. For example, Song et al. [20] proposed a mask-guided background features and pulling body features closer to the full image. Qi et al. [21] used the mask image together with the raw image as inputs and generated fusing features from different levels. Although these methods used mask information, they did not pay enough attention to masks. Therefore, there is still room for improvement using body masks.

### 2.2. Segmentation Models

Segmentation has made great progress in recent years. Many methods based on deep learning [10,11,22] were proposed and achieved state-of-the art results on large segmentation datasets like COCO [23] and PASCAL VOC [24]. In general, current instance segmentation methods are categorized into two types, depending on starting from either detection or segmentation modules. Detection-based methods, such as Faster R-CNN [25], R-FCN [26], obtain the region of predicted boxes, and then predict the mask for each region. Pinheiro et al. [27] proposed DeepMask and Dai et al. [22] proposed instance-sensitive FCNs for generating mask proposals. FCIS [28] used fully convolutional network and considered position-sensitive maps with inside/outside scores. He et al. [10] proposed Mask R-CNN and achieved impressive performance by adding a segmentation branch. Methods based on segmentation conduct pixel-level predictions first and then group them together to obtain segmentation results. Some work [29,30,31] use metric learning to ensure that pixels from the same instance have similar embedding for better segmentation. In addition, some other works [32,33] suggested adding boundary detection information in the second stage. By virtue of these segmentation methods, more accurate masks can be achieved. Thus, combining segmentation and person ReID becomes a new way to obtain body regions explicitly. Qi et al. [21] designed two branches, which use both raw and masked images as inputs, while Song et al. [20] concatenated them to become a single image. However, due to huge difference between segmentation and ReID datasets in resolution, image size, and object classes, body mask generating faces many challenges. Sometimes incomplete masks are segmented and they are usually discarded, which could make the mask-based learning less meaningful and difficult to adapt to changeable situations.

### 2.3. Loss Function 

Another key part for person ReID is how to match the same person after extracting features. Loss functions are designed for guiding the training process. Models for loss functions can be roughly divided into three categories according to different losses. The first one is based on ranking. These methods regard person ReID as a ranking problem based on similarity measurements. Triplet loss and its improvement versions [34] are widely used. Their main idea is to reduce the distance between the same identity so it will be closer than different identities. The second one is using classification. Several approaches treated the ReID as a multi-class classification framework with each person representing a class. For example, Xiao et al. [18] combined multiple datasets and proposed a dropout algorithm using classification network. Yao et al. [4] clustered saliency regions and designed a classify function for each region. Similarly, Sun et al. [35] produced a global classifier at first, split the raw image into six parts, and then connected each part to a classifier. These models show great advantages in the convergence speed and the generalization ability with less possibility of overfitting. The last category applies multi-task methods. For example, Chen et al. [36] combined the ranking loss for features from low levels and the classification loss for features from high levels into the simultaneous training process, and McLaughlin et al. [37] combined verification and classification together and trained them separately for better representation and generalization on unseen datasets.

## 3. Methodology

This section describes the proposed dynamic part attention (DPA) model for person ReID. The overview architecture is shown in Figure 3. It is a two-branch framework. The first is the global branch applying ResNet-50 [38] as the backbone to extract global features, and then uses global average pooling (GAP) to calculate the global loss. The second is the part-attention branch, which is used to extract features of the specific body region and calculate the part loss. Two branches are interrelated but learning features separately, and losses are combined dynamically during training in order to achieve mutual promotion.

### 3.1. Mask Acquisition

In person ReID, background is a major interference factor because pedestrians are prone to be occluded by various objects outdoors such as pillars, vehicles, trees, and so on. Sometimes, a person only occupies a small proportion of an image, and only parts of the body like upper-body or legs are shown in the picture. The DPA model is based on varied body masks, which could dynamically change in shapes and the proportion of person in whole image, representing the attention parts we need to focus.

First, we use segmentation method to extract the body mask from the whole image. Mask R-CNN [10] is a state of-the-art and widely applied segmentation method with competitive performance in large-scale datasets, combining multiple tasks including object instance segmentation, object detection, and key point detection, so we employ this auto-segmentation method for our person mask acquisition instead of manual labeling. As shown in Figure 3, Mask R-CNN [10] is a detection-based segmentation method and it consists of two stages. The first stage is to propose candidate object bounding boxes regardless of object categories. The second stage is to extract features for each proposal and performs proposal classification, bounding box regression and mask predicting. We use RGB images as inputs and after applying Mask R-CNN [10] to obtain person masks, two types of outcomes could be obtained as shown in Figure 2: (a) Complete masks which cover the majority of the body region; and (b) incomplete masks which only cover some parts of bodies.

The reasons for incomplete masks are partly because some pedestrian images are in low resolution, and partly due to biases between training datasets of segmentation and person ReID. However, for incomplete masks, it is worth noticing that those parts might still have stronger discernibility than other regions. This motivates us to introduce a dynamic part attention mechanism based on masks, which has a good response to variable situations.

To elaborate our idea, we first extract a binary mask, which is made up of 1 or 0 for every pixel in the original image, with 1 denoting that the pixel belongs to person and 0 the non-person background. To distinguish intensity of attention paid to masks, we design a margin λ as the basic of calculation. The margin λ indicates the ratio of pixels with value 1 to the whole image.

(1) $$\lambda =\frac{\sum_{i}^{n}({\mathrm{pixel}}_{i}=1)}{\mathrm{wh}}$$

where *w* and *h* are the width and height of the image, and *pixel_i_* is the value of *pixel_i_* among *n* pixels in the whole binary mask image.

### 3.2. Global Description Branch

To extract comprehensive features and avoid missing some useful information, we introduce a global branch and select the backbone of network from typical CNN networks, such as ResNet [38] and Google Inception [39], which are designed for classification and pre-trained on ImageNet dataset [40]. Note for each specific application, the last fully connected layer is replaced, and basic parameters are fine-tuned through training process. In this paper, we choose ResNet-50 [38] as the backbone to keep a relative balance between performance and complexity. We then model the person ReID as a multi-class classification work. In most cases, a fully connected layer is designed after several convolutional layers to integrate learned features for classification. However, recent research suggested replacing the fully connected layer by a Global Average Pooling (GAP) layer [41] due to redundant parameters and a high possibility of overfitting [4,36]. Therefore, we use a GAP layer as described in the following equation:

(2) $$F_{c}=\frac{1}{\mathrm{wh}}\sum_{i=1}^{w}\sum_{j=1}^{h}f_{\mathrm{ij}},$$

where *F_c_* is the value of channel *c* after the GAP layer, *w* and *h* are the height and width of the image, *f_ij_* presents the pixel value in location of width *i* and height *j.* The GAP layer takes average of the last feature maps in each channel. We then use Softmax activation and Cross-Entropy loss to measure the error:

(3) $$L_{\mathrm{global}}=-\sum_{i=1}^{N}\log\frac{\exp(W_{y_{i}}^{T}x_{i})}{\sum_{j=1}^{k}\exp(W_{j}^{T}x_{i})},$$

where *L_global_* is Cross-Entropy loss of the global image, *i* is the index of the image, *x_i_* is the input feature of *i*-th sample, *y_i_* is the identity of *i*-th sample, and *N* is the total number of images in case of batch-training, *k* is the number of person identities, *W_j_* is the classifier for *j*-th identity. Softmax function is used in the multi-classification process and it maps the output of multiple neurons to the (0,1) interval and output the probability to make multi-classification. *L_global_* can be understood to measure the classification ability of the model based on predicted results.

### 3.3. Dynamic Part-Attention Branch

The body attention part, based on segmented mask, called mask attention part in following contents. Usually masks are introduced into network as apart of inputs stacking or being independent of original RGB images, leading to additional and repeated calculation on low-level features. With the faith of focusing on partially salient features of bodies without ignoring useful global information, we propose a succinct network using only original images as inputs and adding masks subsequently. When it comes to introduce masks information, we have two considerations, as follows:

**• First, how to introduce masks?**

We want to establish a better correspondence between global and mask attention part. Therefore, instead of extracting features separately, we select attention parts based on global feature maps and binary masks. Specifically, we can obtain feature maps through consecutive learning of the CNN network. So, we extract feature maps after a specific layer; resize binary masks into the same size of extracted feature maps, and then map binary masks that only contain values of 0 or 1 to feature maps to filter redundant information and get features of mask attention part called Mask-maps as:

*R*_*i*,*j*,*c*_ = *F*_*i*,*j*,*c*_·*M*_*i*,*j*,*c*_, (4)

where *R*_*i*,*j*,*c*_ is the pixel value of Mask-maps at the location of corresponding width, height, and channel, *F*_*i*,*j*,*c*_ and *M*_*i*,*j*,*c*_ denotes the similar meaning of the feature maps and binary masks. Then we obtain salient Mask-maps based on global features and masks in the network.

**• Second, where to introduce masks?**

As demonstrated above, we select feature maps after a specific layer for mapping, but which layer should we choose is worth considering. There is a gap in spatial shape and semantical information between features from various hierarchies [42]. Low-level semantic features extract specific and simple characteristics, while high-level targeting on abstract features but less location information. Our method of Mask maps selection needs to achieve a balance between discriminative features and location information form aping properly. At last, we select the feature maps *δ* after the first convolutional layer through experiments. We will discuss the detailed selection in Section 4.3.

After obtaining Mask-maps consisting of salient body features based on masks, we bring them to another branch for learning and description. Similar to the global branch in Section 3.2, we apply GAP to Mask-maps to get a fixed sized vector and then connect it with the Cross-Entropy loss directly as the mask-part loss:

(5) $$L_{\mathrm{mask}}=-\sum_{i=1}^{N}\log\frac{\exp(W_{my_{i}}^{T}\;x_{\mathrm{mi}})}{\sum_{j=1}^{k}\exp(W_{\mathrm{mj}}^{T}\;x_{\mathrm{mi}})},$$

where *mi* is the index of masks, *x_mi_* is the input feature of masks, *k* is the number of person identities, *W_mj_* is the classifier for *mj*-th identity for another classification, and *N* is the total number of images. It is similar to Equation (3). This not only avoids disadvantages of FC-layer in overfitting and oversize parameters, but also makes the model more compact and consistent with the global branch.

### 3.4. Dynamic Loss Combination 

It is indicated in Reference [43] that background can provide some context information in the scene, so we integrate background and foreground information together. In particular, we combine the global classification loss and the part attention classification loss as the total loss in our model as shown in Equation (6):

(6) $$L_{\mathrm{total}}=\alpha \;L_{\mathrm{global}}+S(\lambda )\;L_{\mathrm{mask}},$$

(7) $$S(\lambda )=\frac{1}{1+e^{-\lambda }},$$

(8) α+S (λ)=1,

where *α* is the proportion of global loss, and *λ* is undertaken from Section 3.1 indicating the ratio of pixels with value 1 to the whole image. The margin *λ* denotes attention degree we put on complete masks. The influence of each loss on the combined one can be controlled through parameter α and λ in Equation (6). Given the idea of introducing flexible attention degree based on mask ratios, masks with relatively complete segmentation are supposed to obtain higher weights, but incomplete masks deserve attention to some extent due to their more discrimination than others. So, we introduce Sigmoid function to obtain soft weights of part attention loss based on the mask ratio *λ* in Equation (7), leading to consecutive and undiminished attention on mask parts. We design *α* related with the part-attention loss and set the sum to 1 in Equation (8) to achieve a complementary state at the same time. This dynamic adjustment of weights between global and part-attention branch guides the learning process under both restraints, which achieve mutual improvement in the whole process. It will leave inclusivity for variable conditions and make our network more powerful as a feature descriptor.

## 4. Experiments

### 4.1. Datasets and Protocols

We verify the proposed method in three public datasets: Market-1501 [44], DukeMTMC-reID [45], CUHK03 [46], and each of them is a large-scale ReID dataset containing more than 1000 identities:

Market-1501: The Market-1501 contains 32,668 annotated bounding boxes from 1501 identities captured by six cameras, in which 751 identities were used for training and 750 identities for testing. Each identity is consistent in several images captured from at least two cameras, which were selected by the overlap ratio between manually annotations and the person detector DPM [47]. In the testing set of 750 identities, for each identity, only one image from each camera is selected as a query, resulting in 3368 query images in total, while the rest are gallery images. 

DukeMTMC-reID: It is one of the largest pedestrian datasets which consist of 1404 identities of 36,411 hand drawn bounding boxes. The dataset contains 16,522 images of 702 identities for training, 2228 query images, and 17,661 gallery images of the other 702 query identities for testing. The identities are randomly split into the training set and the testing set in equal halves. All images are captured from eight cameras and many pedestrians are in similar appearance, so it is full of challenge. 

CUHK03: This dataset is composed of more than 14,000 images of 1467 identities collected from five pairs of cameras in the CUHK campus, in which 767 identities for training and 700 for testing. There are two parts: In the detected set, all the bounding boxes are detected by the person detector DPM [47] detection method; in the labeled set, boxes are drawn manually for better accuracy. We conduct experiments on both types of annotated datasets.

Protocols: Following the standard evaluation protocols, we adopt the cumulative matching characteristics (CMC) at Rank-1 identification rate and mean average precision (mAP) for evaluation. The two values can reflect the performance more comprehensively in both precision and recall rate.

For better verification of practical effectiveness, we also test our model on the dataset collected by ourselves. We provide two practical scenarios. The first is video data collected from an intersection with pedestrians under crowded condition, which contains 10 signal cycles of the pedestrian phase. The second video data is collected from a moving robot with moving angles in 15 min and records situations when following a walking man with occlusion, target missing, etc.

### 4.2. Implementation Details 

We apply ResNet-50 [38] as the backbone network, use the initial model pre-trained on ImageNet [40], and replace the last fully connected layer with a GAP and a classify layer in both global and part-attention training.

For each image, we use Mask R-CNN [10] to get body masks. Masks are changeable due to variable scenes and even a proportion of images fail to get satisfying masks. Therefore, we propose a dynamic part-attention model based on masks to respond to different cases. Inspired by Reference [35] and through many experiments, we resize images into the fixed size of (384,128), and then employ the data augmentation including normalization and horizontal flip randomly. For three datasets, we train the model for 60 epochs with the batch size of 32 on GTX TITAN X GPU. The optimizer is Stochastic Gradient Descent (SGD) and the initial learning rate is set to 0.01, which is decayed after 20 epochs with a gamma of 0.01.

### 4.3. Effectiveness Analysis

We evaluate effectiveness of the proposed method mainly on the widely used Market-1501 dataset. The following presents four parts of comparison.

**• The effect of the part-attention branch.**

To first verify the influence of the part branch using segmented masks, we compared the baseline model similar to Reference [35] containing global branch only. The baseline chooses ResNet-50 [38] as backbone and discards the last pooling layer and dropout layer. Compared with the baseline, our method achieves 4.42% improvement in rank-1 accuracy and 5.33% in mAP as shown in Table 1. This confirms effectiveness of part attention branch based on variable masks.

**Figure 4:**
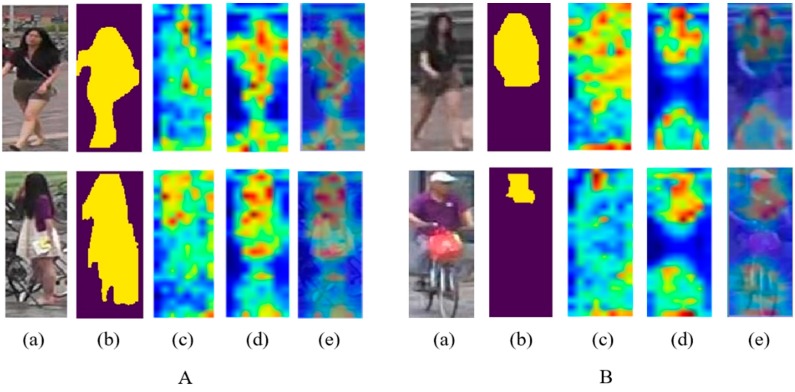
Comparison of saliency maps between different methods. Group A denotes images with relatively complete masks, while group B are with incomplete masks. In both A and B, (**a**) and (**b**) are input RGB images and masks through segmentation. (**c**) to (**e**) are: Saliency maps from traditional baseline method, saliency maps from our method with a dynamic part-attention branch, and overlay results between (**a**) and (**d**) for better visualization. Best viewed on a color screen.

To explore potential reasons of getting the boost, we extract saliency maps of CNN learned in different methods as shown in Figure 4. The traditional baseline method using only global information extracts scattered features including the body and background among the whole image like (c), and this brings obstacles to extract discriminative features due to mutable situations. Conversely, our method that adding dynamic masks attention parts enabling the network to mainly focus on the body like (d), extracts more concentrated features and becomes a powerful descriptor. It is worth mentioning that through continuous learning, the network can target on the body region consciously even with in complete masks like group B in Figure 4, which enables our method to respond to variable situations.

**• Mapping layer selection.**

Features extracted from different convolutional layers contains semantic information of different hierarchy. We choose feature maps from one layer in global branch to map binary masks and obtain attention Mask-maps. In order to verify the specific time for mapping masks, we extract feature maps after different layers from the network to conduct experiments. Additionally, this comparison includes mapping masks on the original images called RGB-mask layer, which is equivalent to two branches learning with one branch of original images and another of partly RGB images containing mask regions. As shown in Table 2, layer names are related with the ResNet-50 [38] backbone. Conv1 represents mapping masks after the first convolutional layer, while Layer1 to Layer4 represent four block structure in ResNet-50 [38]. It indicates that mapping at Conv1 layer leads to higher Rank1 while higher layer performs worse, but Layer4 obtains better mAP. It can be inferred that consecutive convolution operation extract more discriminative features, but lose location information, which is important for location correspondence when mapping masks. Relatively accurate location and the most discriminative features are two key factors in our part attention learning.

**• Dynamic loss proportion.**

During the training process, we combine losses of two branches dynamically in Equation (6). The mask attention part loss is based on masks ratio and influence the global proportion directly. In Equation (8), we set their sum to 1 to achieve a bilateral complementary state.

To verify effectiveness of this dynamic setting, we also fix the proportion of the global and part loss for experiment. Under the limit of Equation (8), we set the ratio between global and part loss in five groups and compare with primary dynamic setting in Table 3. It shows that the initial dynamic setting performs better than rigid proportion, which proves the dynamic integration of global and mask information and shows that restricting each other can strengthen the ability of our network. Relatively lower global proportion leads to worse performance, so the global branch plays an indispensable role in the integrated and complementary process.

**• Another parameter analysis.**

A typical CNN network like ResNet-50 [38] was chosen as the baseline is pre-trained on ImageNet with the input image size of 224 × 224. However, datasets of person ReID usually have rectangle shapes, including 128 × 64, 256 × 128 or diverse sizes in a single dataset. The image size is an important parameter because it affects the output size of feature maps directly. Therefore, we select several kinds of image sizes to explore the specific influence of shape as shown in Table 4. It shows the influence of the spatial size and the ratio simultaneously. The trend indicates that a larger size and ratio can benefit the ability of representation, but there is a limit for keeping the consistency and reality of images.

### 4.4. Comparison with Other Methods

To verify effectiveness and generalization of our method, we compare results with the state-of-the-art methods on three popular datasets. We evaluate the performance with Rank-1 and mAP. We also employ the re-ranking method [48], which is commonly accepted in person ReID. 

**Market-1501**: We choose several state-of-the-art methods for comparison and the results are shown in Table 5. For single query, our method achieves 89.72% Rank-1 accuracy and 73.83% mAP, outperforming most of other state-of-the art methods. With the help of re-ranking, the Rank-1 accuracy and mAP reach to 91.68% and 88.29%, respectively. For multiple query, our method achieves 94.14% Rank-1 accuracy and 90.31% mAP, exceeding other methods too. Note that our model does not any pre-training, which indicates our method is more robust and effective.

**DukeMTMC-reID**: The comparison with several state of-the-art methods are summarized in Table 6. In single query, our method achieves 79.7% Rank-1 accuracy and 62.1% mAP in single query, outperforming the compared methods. After re-ranking, the performance is promoted to 84.2% Rank-1 accuracy and 78.8% mAP.

**CUHK03**: We evaluate the proposed method using the new evaluation protocol in Reference [49] on both detected and labeled datasets. Rank-1 and mAP are compared with recently state-of-the art methods in single query. As shown in Table 7, we achieved competitive results in two datasets, and after re-ranking, we further boost results to 62.39% Rank-1 accuracy and 60.57% mAP in detected dataset, and 63.04% Rank-1 accuracy and 61.73% mAP in labeled dataset. Note that this new protocol uses a larger testing gallery, which is more challenging and showing effectiveness of our method.

Furthermore, the model is trained from scratch without any pretraining. In spite of biases in scenes and detection variance between three datasets, our method arrives at competitive performance on all datasets, indicating both robustness and effectiveness.

### 4.5. Practical Applications with Own Datasets

In recent years, person Re-ID has drawn an increasing interest in both academia and industry due to its great potential in video surveillance applications, human-computer interaction, robotics, and content-based video retrieval. 

**Figure 5:**
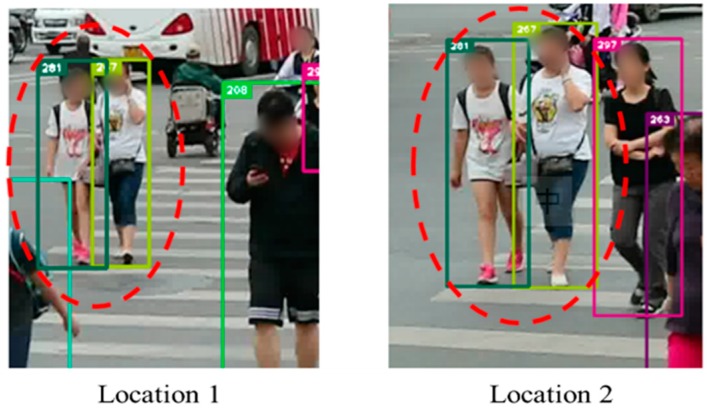
The effectiveness of result on video from intersection. Our method is applied to help identify target persons and track moving trajectories of pedestrians.

To verify the practical effectiveness of the proposed model, we further test the method by using video data collected from an intersection with multiple persons under crowded condition. The model can be applied in multiple object tracking to help identify persons and follow their moving trajectories with high accuracy. As shown in Figure 5, our model can keep identifying targeted persons and we can obtain continuous pedestrian trajectories. We use ID-switch to measure the performance which means that the targeted identity number has been transformed during the process. The video contains 10 signal cycles of the pedestrian phase, and we sum up the total number of ID-switch. As shown in Table 8, compared with the tracking method without our ReID model, we decrease 23 times of ID-switch, which means reducing failing by 41% under the same crowded condition. So, the improvement is significant in practical applications. 

**Figure 6:**
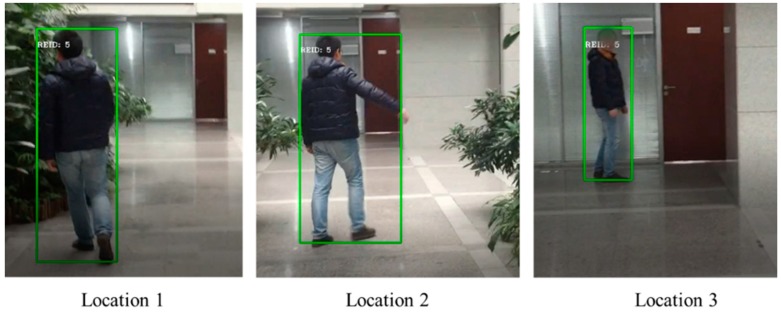
A long period tracking in the view of robotics with DPA model. Our method is applied to help keep following the target person for a long period with interference and occlusion.

We further test our method for the data collected from videos with different angles and heights. The video was installed in a moving robot with moving angles and heights. During the test, a robot can follow and identify the target person by giving with the same ID for a long period under the environment with occlusion, multi-person interference, changes of angles, etc. As shown in Figure 6, our method can be applied to keep identifying and matching the target person successfully regardless of changes of angles and heights. Compared to the baseline model mentioned in Section 4.3, our method decreases the ratio of ID changes (i.e., failure of tracking target) by 10%.

For computation speed, our method achieves 50 FPS in GTX TITAN X GPU during testing. We further implemented the whole method on embedded device after engineering acceleration, and the proposed method achieved the real-time speed of 20 FPS. Therefore, it is safe to claim that the proposed model can be applied for real-time applications. However, it should be noted that at current stage of our research, we have not applied our models for any real commercial applications.

## 5. Conclusions

In this paper, a novel dynamic part-attention (DPA) model based on masks for person ReID is proposed. In this model, two complementary classification branches are designed to extract global and dynamic partial features during training. Two branches are not isolated and the part attention branch is based on the global branch. We introduce a dynamic combination of two branches to perform as a comprehensive, but targeted, descriptor, which achieves a mutual and complementary state, further enhancing the over ReID performance. Results of experiments and comparison have confirmed the effectiveness of the proposed network. 

In future work, we will expand the current model with the key points of the human body, from a view of combining masks and key points to match body parts precisely. The expanded method is expected to reduce superfluous information and achieve higher accuracy. 

## Figures and Tables

**Table 1:** The performance comparison between baseline and the proposed method.

Methods	Rank-1 (%)	mAP (%)
Baseline	85.30	68.50
Ours	89.72	73.83

**Table 2:** Performance on Market 1501 with mapping on different layers.

Layer Name	Rank-1 (%)	mAP (%)
RGB-mask	88.71	73.11
**Conv1**	**89.72**	73.83
Layer1	88.74	73.31
Layer2	88.01	73.60
Layer3	87.64	71.59
Layer4	89.44	**74.81**

**Table 3:** Performance with different loss proportion.

Ratio (Global: Part)	Rank-1 (%)	mAP (%)
**Dynamic Dynamic**	**89.72**	**73.83**
2:1	88.86	73.21
3:2	88.39	73.05
1:1	88.53	73.71
1:2	88.36	72.04
1:3	87.63	70.68

**Table 4:** Comparison of the different proportion of global loss.

Image Size	Rank-1 (%)	mAP (%)
128 × 64	80.14	58.14
256 × 128	86.31	68.23
224 × 224	87.77	70.32
**384 × 128**	**89.72**	**73.83**
384 × 192	89.07	73.24
512 × 256	87.47	68.55

**Table 5:** Single query and multiple query comparisons on the Market-1501 dataset.

Methods	Single Query		Multiple Query	
	Rank-1 (%)	mAP (%)	Rank-1 (%)	mAP (%) (%)
BOW + kISSME [45]	44.4	20.8	-	-
WARCA [49]	45.16	-		
Siamese LSTM [2]	-	-	61.6	45.3
Gated [1]	65.88	39.55	76.04	48.45
Spindle [6]	76.9	-	-	-
MSCAN [3]	80.31	57.53	86.79	66.70
SVDNet [50]	82.30	62.10	-	-
MultiLoss [51]	83.9	64.4	89.7	74.5
Triplet Loss [52]	84.92	69.14	90.53	76.42
Deep Mutual [53]	87.73	68.83	91.66	77.14
ours	89.72	73.83	93.23	82.45
Ours + Re-ranking	**91.68**	**88.29**	**94.14**	**90.31**

**Table 6:** Single query comparisons on the DukeMTMC-reID dataset.

Methods	Rank-1 (%)	mAP (%)
BOW + kISSME [45]	25.13	12.17
LOMO + XQDA [54]	30.75	17.04
GAN [45]	67.7	47.1
PAN [9]	71.6	51.5
SVDNet [50]	76.7	56.8
MultiScale [16]	79.2	60.6
Ours	79.0	62.1
Ours+Re-ranking	**84.2**	**78.8**

**Table 7:** Single query comparisons on the CUHK03 dataset.

Methods	Detected		Labeled	
	Rank-1 (%)	mAP (%)	Rank-1 (%)	mAP (%)
BOW+XQDA [44]	6.36	6.39	7.93	7.29
LOMO+XQDA [54]	12.8	11.5	14.8	13.6
PAN [9]	36.3	34.0	36.9	35.0
SVDNet [50]	41.5	37.26	40.93	37.83
HA-CNN [55]	41.7	38.6	44.3	41.0
MLFN [56]	52.8	37.8	54.7	49.2
Ours	53.04	48.32	54.27	49.96
Ours + Re-ranking	**62.39**	**60.57**	**63.04**	**61.73**

**Table 8:** The performance comparison when our model is applied to multiple object tracking.

Methods	ID-Switch	Improvement
Track only	56	-
Track + ours	33	41%
